# Single Fusion Image from Collections of Fruit Views for Defect Detection and Classification

**DOI:** 10.3390/s22145452

**Published:** 2022-07-21

**Authors:** Antonio Albiol, Carlos Sánchez de Merás, Alberto Albiol, Sara Hinojosa

**Affiliations:** 1 Departamento de Comunicaciones, Universitat Politècnica de València, 46022 Valencia, Spain; carsande@doctor.upv.es; 2 ITEAM Research Institute, Universitat Politècnica de València, 46022 Valencia, Spain; alabiol@iteam.upv.es; 3 Multiscan Technologies SL, 03820 Cocentaina, Spain; shinojosa@multiscan.eu

**Keywords:** 3D, rotation, mapping, projection, unwrapping, quality assessment, fruit

## Abstract

Quality assessment is one of the most common processes in the agri-food industry. Typically, this task involves the analysis of multiple views of the fruit. Generally speaking, analyzing these single views is a highly time-consuming operation. Moreover, there is usually significant overlap between consecutive views, so it might be necessary to provide a mechanism to cope with the redundancy and prevent the multiple counting of defect points. This paper presents a method to create surface maps of fruit from collections of views obtained when the piece is rotating. This single image map combines the information contained in the views, thus reducing the number of analysis operations and avoiding possible miscounts in the number of defects. After assigning each piece with a simple geometrical model, 3D rotation between consecutive views is estimated only from the captured images, without any further need for sensors or information about the conveyor. The fact that rotation is estimated directly from the views makes this novel methodology readily usable in high-throughput industrial inspection machines without any special hardware modification. As proof of this technique’s usefulness, an application is shown where maps have been used as input to a CNN to classify oranges into different categories.

## 1. Introduction

The inspection of fruit and vegetables is of paramount importance in food quality assessment. For this reason, many different methods and techniques have been developed and used in this area [1,2,3], with computer vision being one of the most cost-effective solutions.

The exploration of the whole fruit surface requires several pictures from different directions. This can be carried out using conveniently distributed cameras [4,5,6,7,8] or a single camera. In this latter case, the different views of the object can be obtained with various solutions such as mirrors [9,10,11,12] or by rotating the fruit [13,14]. However, the use of mirrors is not suitable because dirt or dust can be deposited on its surface during the continuous operation of the machine.

**Figure 1:**
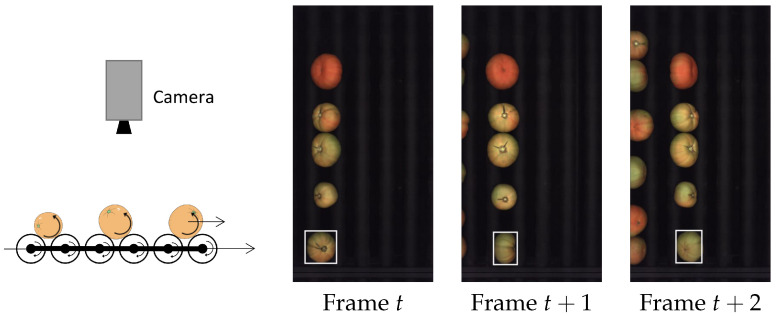
Roller conveyor unit used to obtain the different views of the rotating fruit and three consecutive camera frames. The fruit rotate while traveling to the right.

In this work, a roller conveyor unit of an industrial inspection machine has been used to obtain the different rotated views of each fruit traveling under the camera. In this machine, it is possible to adjust the rotation speed independently of the linear traveling speed. A diagram of the method to obtain views from different directions of each fruit is depicted in Figure 1. The camera is pointing downwards, perpendicularly to the traveling direction of fruit. The camera is oriented in such a way that the piece of fruit captured by the camera travels horizontally and rotates around a vertical axis. In these inspection machines, fruit can travel and rotate at high speeds. In about 1 s, the fruit turns about $360^{\circ }$ and leaves the camera’s field of view, achieving throughputs of about 20,000 kg/h of fruit.

An example of the kind of camera view is shown in Figure 1, where the vertical rolling bars that rotate the fruit while transporting them can be appreciated. A collection of views of the same fruit is obtained by tracking each object.

Grading the quality of a piece of fruit involves taking a global decision from the analysis of this collection of views. The decision is based on the number and severity of defects.

Having a collection of views for each fruit rather than a single image poses several challenges, namely:

- For small area defects, a mechanism that prevents counting them multiple times in different views is necessary.
- For extensive defects, they may not be entirely seen in any single view.
- Measuring global features, such as the fraction of ripe surface, is difficult due to the overlap of views.

This paper’s primary goal is to create a single image, denoted as the map, which integrates the information of all the views. The map should help to grade the fruit, at least for the original views, without combining data from the analysis of the individual views.

The paper’s novelty lies in the fact that this is the first time (to the best of our knowledge) that a synthetic image that integrates the information from a collection of views is proposed to grade fruit. In Section 1.1, we will provide more details about other works that address the problem of grading fruit from collections of views.

This paper can be considered an extension of our previous work [15] about how to estimate the 3D rotation between consecutive views of fruit by assuming a spheroidal model. Rotation estimation from the views is crucial since the assumption of slip-less rotation fails in the case of real fruit. The central idea in [15] was to estimate the rotation between consecutive views in order to predict the position of a point of one view in the next one. On the other hand, the work presented in this paper relies on those rotation estimations in order to synthesize a single image which represents the whole surface of the fruit.

Because of the fruit geometry, it is impossible to create a flat image of the surface with no geometric distortion. The created map will be a Mercator-like projection of the fruit surface, whose two dimensions correspond to longitude (horizontal) and latitude (vertical). We will choose the pole–pole axis to be aligned to the fruit rotation axis so that most of the content of the views will be close to the equivalent of the earth equator on the map, where geometric distortion is the smallest.

Our method can be applied to fruit with approximately spheroidal geometry, such as tomatoes, mandarins, oranges, peaches, etc. Fruit that does not fit this model, such as bananas, avocados, pears, etc., are out of the scope of this paper.

**Figure 2:**
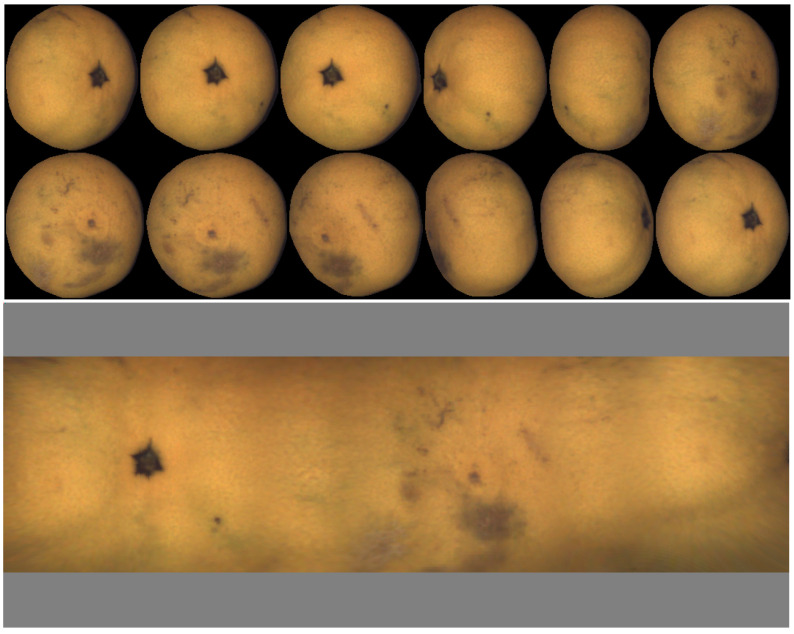
Set of views and resulting map.

An example of a map of a mandarin, together with the set of views, is shown in Figure 2. The gray bands on the top and bottom of the map correspond to areas not explored in the views close to the rotation axis (equivalent to polar earth regions).

To be useful for defect analysis, the map must have the following properties:

- Lossless: The quantity and quality of visual information in the views should be retained on the map. All the analysis that can be conducted on the views should be possible on the map.
- Seamless: The map should not exhibit discontinuities at the transitions between views and contributions.
- Efficient computation: Each point on the fruit surface may appear in a relatively high number of views (about four in the example of Figure 2). Any point in the map should be computed based on two views at most to decrease the computational cost. The two views that contribute to each point of the map are selected as those where the point of the fruit appears closest to the view center, that is, where geometric distortion is the lowest.

One difference between our maps and earth maps is that earth maps have a longitude span of $360^{\circ }$. In our case, depending on how much each fruit has rotated, it is possible to have maps with less than $360^{\circ }$ of longitude span when the fruit has not completed one complete turn and maps with more than $360^{\circ }$ of longitude span if the rotation exceeds one full turn. In the first case, it is possible to assess the idea that only a fraction of the fruit has been inspected. In the latter one, features that appear in the first views reappear in the last ones. This is not a problem for defect analysis since it is known that two points $360^{\circ }$ apart on the map correspond to the same on the fruit surface.

All the maps shown in the paper are created, starting with the first view until the last one. Information on first views appears on the left part of the map. Final views are represented on the right part of the map.

The rest of the paper is organized as follows. Section 2 offers a detailed description of the method to create the maps. Section 3 shows examples of fruit maps generated using the proposed method. Section 3.2 presents an application of the generated maps. We used orange maps to train a CNN that classifies them into eight types of defects.

Finally, we derive some conclusions and present some possible extensions of this work.

### 1.1. Previous Work

We have not found any reference that presents a method to create a surface map from collections of views of images obtained with area sensors. In [16], a system that uses a hyperspectral line sensor is described. They rotate the fruit using rollers that rotate very slowly (compared to our acquisition machine), and lines are captured at a rate of 200 lines per second (much higher than ours) to obtain a 2D image from the line sensor. The obtained 2D image is very similar to our maps. The critical difference with our proposal is that they only need to stack the lines to create the map. Since their primary concern is to demonstrate the utility of hyperspectral information to assess peach quality, the way images are acquired has a secondary role. No emphasis is placed on the time to record the data. The use of line cameras in industrial inspection machines is not an alternative because the fruit must travel as they rotate. In the setup of this paper, the fruit was at a fixed placement and just turned under the line sensor.

In [13], a similar imaging system with a hyperspectral line camera is used, and scanning is performed either under linear movement or rotation. For this latter case, specific conditions are set to keep the distortion minimum. Again, their primary interest is not the image in itself but the application of hyperspectral imaging, and no information about capturing time is presented.

In [17], the authors propose a method, limited to spheres, that uses a pair of stereo cameras to obtain six (2×3) views of a spherical object. They assume that the object rotation is slip-less and that the true rotation is known given the diameter of the sphere. This reference aims to obtain zones of responsibility in the different views so that all the zones cover all the fruit surface and there is no overlap between them. The ultimate objective is similar to ours: to prevent duplicate defects in more than one view. However, they do not attempt to create a synthetic image; they are restricted to spherical geometry and assume that the rotation is known.

The only reference that does something similar to using a single image to grade the whole surface of a fruit is [18]. The main goal of this reference is to describe an approach to inspecting citrus. To prevent the problem of partial information in the views, they propose the creation of a new image composed of a central window of each of the views. These strips are just appended one next to the other. Again, the size of the strip, which should depend on the rotation speed, is estimated by the use of encoders and under the assumption of slip-less rotation. The resulting image has the appearance of a collage of image portions.

None of the other references consulted [9,10,11,12,14] that use different views of the fruit to explore the whole surface create any kind of single image representation. All of them analyze the views one by one.

Finally, another computer vision topic related to the research of this paper is image stitching [19], where a panoramic image is created from collections of pictures. One essential difference between image stitching and this paper is that the former assumes that the camera position does not change between the individual pictures; thus, a homography is a good approximation for the relation between views. However, in our case, the relative position between camera and fruit changes as the fruit rotates and the relation between the contents of the views is no longer a homography.

## 2. Materials and Methods

This section will provide a detailed description of how the map is constructed.

### 2.1. Geometry Modeling and Rotation Estimation

**Figure 3:**
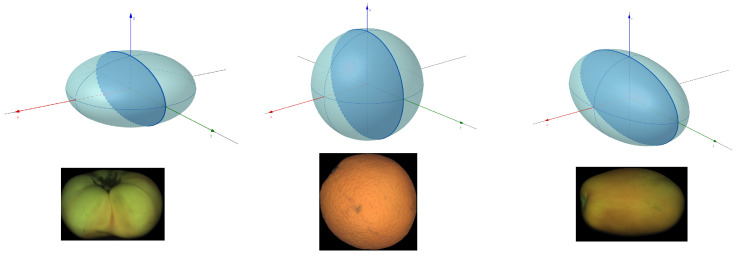
Fruit geometry models and examples. From left to right: oblate, spherical, and prolate models.

To build the map, it is necessary to estimate the rotation between consecutive views and a geometric model of the fruit. Fruit is modeled as spheroids (ellipsoids of revolution). For clarity, we reiterate that a spheroid is just an ellipsoid with two of the three radii being the same size. Figure 3 shows the three spheroid models covered in this work and examples of fruit.

For this purpose, we followed the method described in our previous work [15]. Several conditions must be fulfilled for this method to work correctly:

- The fruit must exhibit some texture so that rotation is observable in the images.
- The rotation between consecutive views must be small enough to allow a significant overlap of content with successive views. This is necessary to estimate the rotation. Rotation angles in the range of 20–$40^{\circ }$ between consecutive views are successfully used.

The outcome of this method (that will be used as input to create the map) is:

- The lengths of the three principal axes of the fruit. Depending on the model (spherical or not), the three axes may have the same size, or one of them will be different.
- The 3D rotation between consecutive views, expressed as a rotation matrix.

Two coordinate systems will be used:

- View-centric coordinate system. Each view will have its coordinate system, with the *x*-axis being the horizontal coordinate, the *y*-axis being vertical, and the *z*-axis being orthogonal to the image plane. The origin of coordinates will be at *the center of mass* of the fruit. z>0 means that the point is above its center of mass, or in other words, that the point is visible.
- Fruit-centric coordinate system. This is a coordinate frame that is rigidly attached to the fruit. So when the fruit rotates, this coordinate frame rotates too. In the case of non-spheric objects, one of the axes of the coordinate system will be coincident with the *different fruit axis*. Since at least two dimensions are equal, there is one degree of freedom to choose the other two axes.

The fruit axes rigidly rotate with the object, so if they are known for one view, they can be known for any other view by applying appropriate rotation matrices. More specifically, if $v_{x}^{\left(i\right)}$, $v_{y}^{\left(i\right)}$, and $v_{z}^{\left(i\right)}$ denote the fruit axes unit vectors in the view coordinate system at view *i*, the fruit axes at view *j* can be obtained by

(1) $$v_{x}^{\left(j\right)}=R_{i}^{j}v_{x}^{\left(i\right)}\;;\;v_{y}^{\left(j\right)}=R_{i}^{j}v_{y}^{\left(i\right)}\;;\;v_{z}^{\left(j\right)}=R_{i}^{j}v_{z}^{\left(i\right)}$$

where $R_{i}^{j}$ is the rotation matrix between views *i* and *j*. To obtain this matrix from the rotation matrix between consecutive views:

(2) $$\begin{array}{c} \begin{array}{cc} R_{i}^{j}= & R_{j-1}^{j}\cdots R_{i+1}^{i+2}\;R_{i}^{i+1}\;\;j>i\\ R_{i}^{j}= & R_{j+1}^{j}\cdots R_{i-1}^{i-2}\;R_{i}^{i-1}\;\;j<i \end{array} \end{array}$$

where $R_{i}^{i-1}={\left(R_{i-1}^{i}\right)}^{-1}$.

The view where fruit axes are set up will be denoted as the *reference view*. If the object is spherical, the fruit axes are chosen to coincide with the view axes at the first view.

If the object is not spherical, the view where the different axis of fruit is parallel to the image plane is chosen. For example, in the case of oblate fruit (such as mandarins and many varieties of tomatoes), the reference view is selected as the one with the shortest minor axis. In the case of Figure 2, the reference view is the fifth in the top row. The view with the longest major axis is chosen in the case of prolate fruit.

**Figure 4:**
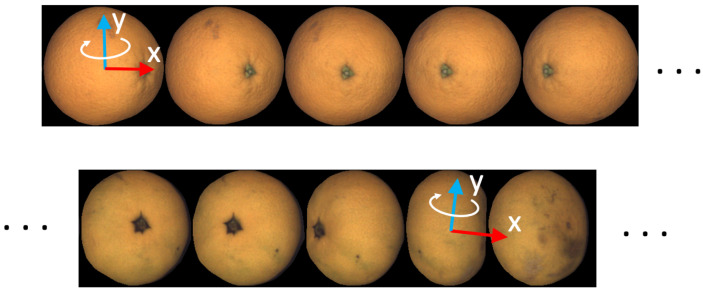
Choice of fruit axes at reference view for spherical (**top**) and oblate (**bottom**) fruit geometry.

The fruit coordinates frame is set up once the reference view is selected. The *y*-axis of fruit is chosen as close as possible to the rotation axis. In particular,

- For the case of spherical objects, fruit axes at the reference view are aligned with view axes (see Figure 4). Remember that in our case, because of the camera setup, fruit rotate around an approximately vertical axis.
- For the case of non-spherical objects, the fruit *y*-axis is chosen as the view principal axis closer to the expected rotation axis. The expected rotation axis is vertical following the construction of the machine (rolling bars can be seen in Figure 1). The fruit *x*-axis is parallel to the image plane and perpendicular to the fruit *y*-axis. Notice that in the case of non-spherical objects, the fruit axes at the reference view do not necessarily align with the image axes.

Figure 4 shows how axes are chosen for two geometries. In the case of spherical fruit, the reference view is the first one. The fruit axes are selected as aligned with the view axes. In the case of an oblate fruit, the view with the shortest minor axis is selected as the reference. Fruit axes are aligned with main axes of the fruit at the reference view. The *y*-axis is selected to be the one closest to the expected rotation, which, in this case, is the vertical direction.

The *z*-axis is chosen simply as $v_{z}^{ref}=\left(0,0,1\right)$ for all geometries.

### 2.2. Map to View and View to Map Correspondence

A point on the map, map(ϕ,λ), where ϕ denotes the longitude and λ denotes the latitude, corresponds to a 3D point (X,Y,Z) on the surface of the fruit. The relation between the coordinates and the latitude and longitude is then:

(3) $$\begin{array}{c} X=r_{x}\cos\lambda \cos\phi \\ Y=r_{y}\sin\lambda \\ Z=r_{z}\cos\lambda \sin\phi \end{array}\}$$

where, according to the geometrical model, $r_{x}$, $r_{y}$, and $r_{z}$ are the three principal radii.

One point of the fruit surface, (X,Y,Z) can be observed in many views. To know where it is in the *i*-th view, we compute

(4) $$\left(x_{i},y_{i},z_{i}\right)=X\;v_{x}^{\left(i\right)}+Y\;v_{y}^{\left(i\right)}+Z\;v_{z}^{\left(i\right)}$$

where coordinates $\left(x_{i},y_{i}\right)$ are relative to the center of mass of the fruit at the *i*-th view. $z_{i}>0$ implies that the point (X,Y,Z) in the *i*-th view will be visible.

Values of $z_{i}$ close to zero correspond to pixels near the silhouette of the fruit in the view.

It is also possible to tell where a point visible in the *i*-th view is mapped. We need to construct a 3D vector and project this vector onto the fruit coordinate system. Let $\left(x_{i},y_{i}\right)$ be the view’s 2D image coordinates of the point of interest (relative to the fruit’s center of mass). We can estimate the *height* of this point, $z_{i}$, using the geometrical model as described in [15]. The absolute fruit coordinates are obtained by projecting vector $\left(x_{i},y_{i},z_{i}\right)$ onto the fruit unit vectors at the current view:

(5) $$\begin{array}{c} X=\left(x_{i},y_{i},z_{i}\right)\cdot v_{x}^{i}\\ Y=\left(x_{i},y_{i},z_{i}\right)\cdot v_{y}^{i}\\ Z=\left(x_{i},y_{i},z_{i}\right)\cdot v_{z}^{i} \end{array}\}$$

Once we have (X,Y,Z), it is straightforward to reverse Equation 3 and obtain longitude and latitude.

### 2.3. Longitude Span of a View

As the object rotates, we move along the longitude axis of the map. Each portion of the map will be filled using the contributions of two views (or one at the beginning and end of the map, see below).

To determine which pair of views will be used at a particular longitude, we proceed as follows:

1. Beginning with the first view, we determine the longitude and latitude of the center of mass of the fruit at each view, as described in Section 2.2. Let $L_{i}$ be the longitude of this central point of view *i* for 1≤i≤N, where *N* denotes the number of views.
2. Since the fruit rotates in a specific known direction, the above longitudes must fulfill $L_{i+1}>L_{i}$. In case this condition fails, we add $360^{\circ }$ to $L_{i+1}$.

**Figure 5:**
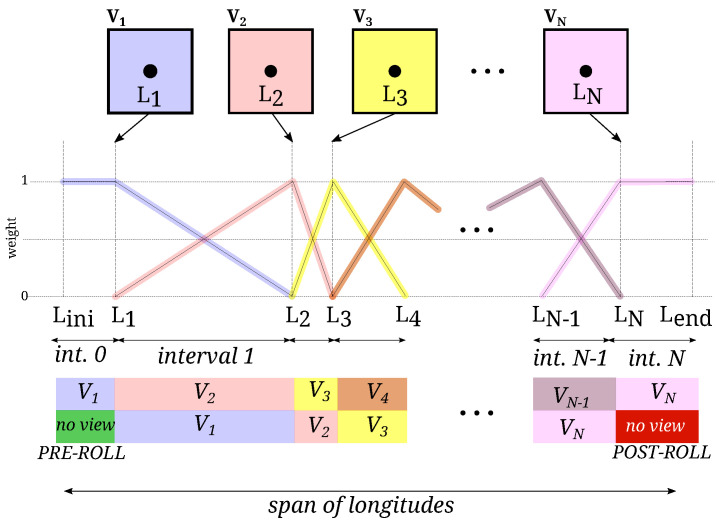
Weight of each view in the composition and contributing views in the longitude range of interest. Notice the extra intervals added before the central longitude of the first view (pre-roll) and after the central longitude of the last view (post-roll). Only one view will be used to draw the map in these intervals.

As a result of the above process, we have an increasing sequence of longitudes $L_{i}$. Figure 5 illustrates the idea of mapping the central point of each view to its corresponding longitude $L_{i}$.

The interval of longitudes between $L_{i}$ and $L_{i+1}$ will be populated using views *i* and i+1, as described below.

**Figure 6:**
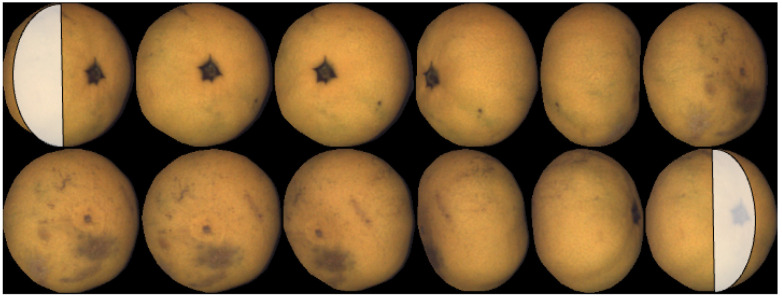
Portion of the first and last view corresponding to pre-roll and post-roll.

To use all the information present in the views, we extend (typically $45^{\circ }$) the map longitude span before $L_{1}$ and after $L_{N}$. These portions of the map are called pre-roll and post-roll. These longitude ranges will be filled using only one view (the first and the last, correspondingly). These regions correspond to the portion of the views shown in Figure 6 and represent the part of the first view that will be occluded when rotating to the second view and the portion of the last view that appears at the end.

### 2.4. Filling the Map

As we have seen in Section 2.3, each longitude of the map will be filled based on the contents of one (pre-roll and post-roll) or two views (general case).

To create a seamless map, the contribution of each view is changed progressively in a linear way between zero and one, as depicted in Figure 5. When the longitude ϕ is between $L_{i}\leq \phi \leq L_{i+1}$, the weights for the *i*-th view, $w_{i}$, and view i+1, $w_{i+1}$, are:

(6) $$\begin{array}{cc} w_{i}\left(\phi \right) & =\left(L_{i+1}-\phi \right)/\left(L_{i+1}-L_{i}\right)\\ w_{i+1}\left(\phi \right) & =\left(\phi -L_{i}\right)/\left(L_{i+1}-L_{i}\right) \end{array}$$

To speed up the process, we do not fill the map for large latitudes since that portion of the map is severely distorted (as in the earth Mercator projections near the poles), and the corresponding points in the views are too close to the silhouette of the fruit.

The whole map creation process can be summarized as follows:

For every latitude λ, such that $\left|\lambda \right|<\lambda_{\max}$, and for any longitude ϕ:

1. Compute the 3D coordinates (X,Y,Z) using Equation (3).
2. Determine the two contributing views for a given longitude ϕ (a single view in pre-roll and post-roll regions) and their corresponding weights using Equation (6).
3. Compute pixel coordinates in both *i* and i+1 views.
4. Calculate the weighted sum of RGB intensities from the two views. Notice that if more spectral channels are available (such as infrared or ultraviolet), this procedure may be extended to them.

**Figure 7:**
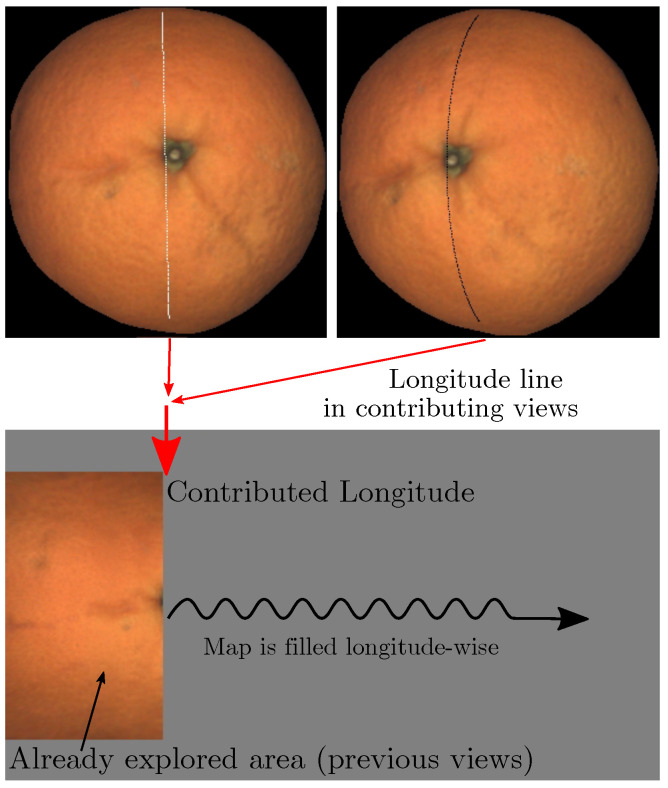
Pixels used to fill one meridian of the map. An animation of the process can be downloaded from [20].

Figure 7 illustrates the process of filling one meridian of the map, and an animation of the mapping process can be found as supplementary material in [20].

The corresponding points in the views computed using Equation (4) are shown. The weight of each view is represented as the color of the arcs in the views. Brighter means a more significant weight. In this case, the first view has greater weight because the arc is closer to the center than in the second view. This figure also allows us to appreciate the fraction of the view that is ignored when limiting latitude to $\lambda_{max}$. The arcs in both views correspond to the same points on the fruit surface.

### 2.5. Considerations about Size and Speed

The size of the map can be adjusted by changing the angular step of one pixel of the map.

The resolution of the map is a critical parameter for the speed of map creation, increasing quadratically as the angular step decreases. The angular step for all the map examples in this paper is chosen so that the resolution of the map at the equator is the same as the resolution of the views at their centers.

Let β be the angular step in radians of the map and *R* be the largest radius of the fruit (in pixels) to obtain a resolution of 1 pixel at the equator. The following relation holds:

(7) βR=1⟶β=1/R

The size of the maps thus depends on the fruit size in pixels. Just as an example, for the case of oranges, there are R≈125 pixels. This yields a map height of about 400 pixels and a map width of 800 pixels.

Typically, the number of views per fruit is between 12 and 15, totaling about 360${}^{\circ }$ of rotation. This can be configured by adjusting the relative speed of translation and rotation motors. The rotation between views cannot be too large to allow for the necessary overlap between views to estimate rotations and cannot be too small since the number of views to explore the 360${}^{\circ }$ range would be too large, increasing computation time.

With these considerations, the whole process of creating the map of one fruit takes about 20 ms on an i7 2021 CPU. This processing time is split into 75% for rotations estimation and 25% for map creation.

## 3. Results

We divided the result section into two parts. Firstly, we will present examples of maps for different kinds of fruit. Since the final goal of the maps is to serve as input for grading methods, we used them to train a convolutional neural network to classify oranges into different categories. Although the paper’s aim is not to present image analysis methods for grading, we thought that this experiment is proof that maps retain the essential information of the collection of views.

### 3.1. Examples of Maps

This subsection presents a collection of maps for different kinds of fruit. The main intention of these examples is to show the quality of the maps and the variation of fruit for which it is possible to generate them.

**Figure 8:**
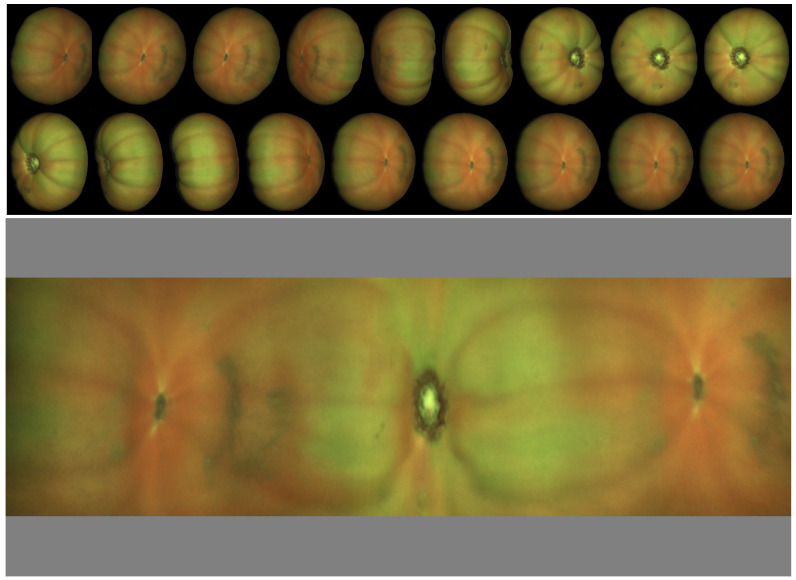
Example of oblate tomato.

A first example corresponding to oblate geometry (mandarin) is presented in Figure 2. One additional oblate example (a tomato in this case) is shown in Figure 8. In this case, it is possible to observe that the tomato rotates more than $360^{\circ }$, and the left part of the map is repeated on the right.

**Figure 9:**
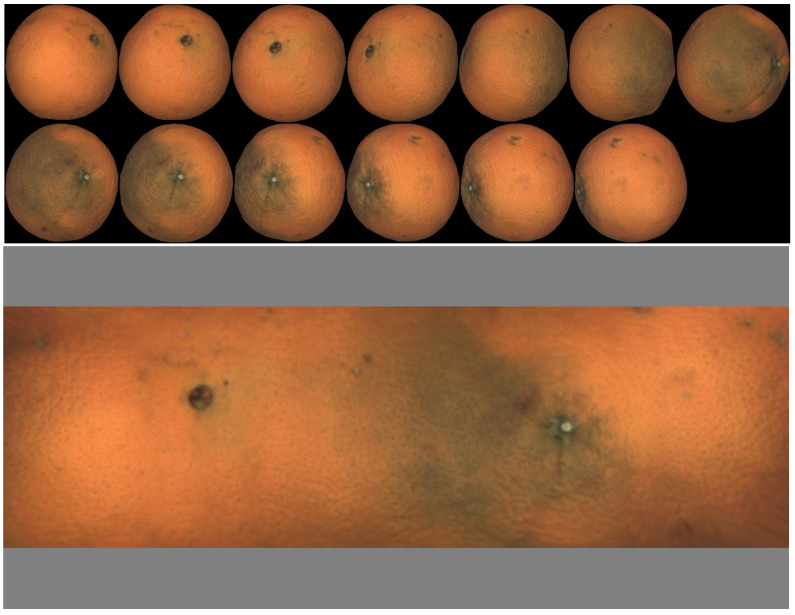
Example of spherical object.

**Figure 10:**
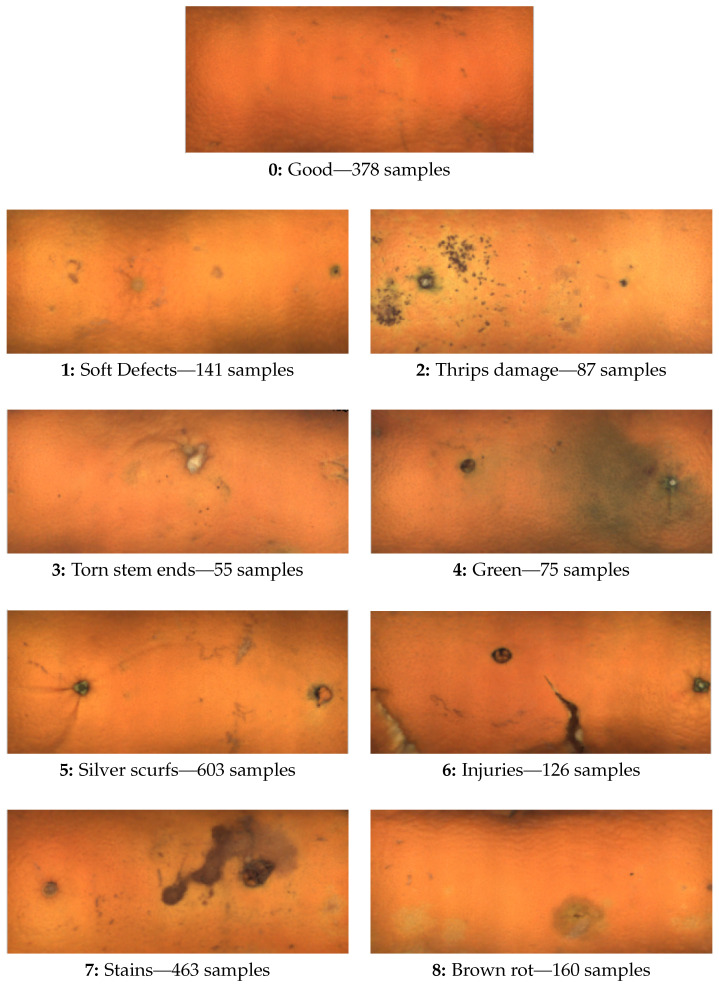
Maps illustrating the different defect categories and the number of samples per category.

In Figure 9, an example of spherical fruit is shown. One problem that is very difficult to solve with view analysis is only evaluating the fraction of green surface of the fruit. From the map, it is straightforward since each map pixel has an associated area depending on its latitude (or latitude and longitude if the geometry is not spherical). This example displays the advantage of analyzing one single image rather than a collection of views. More examples of orange maps are shown in Figure 10.

An additional, more extensive collection of maps can be accessed at [21].

### 3.2. Fruit Classification Based on Maps Using CNN

This section will present one possible application of the maps as samples for training and testing a CNN to classify oranges into different categories.

The classes were defined according to OECD standards [22], and examples of each category are shown in Figure 10.

A dataset was obtained in an industrial inspection machine with various examples (a collection of 14–16 views corresponding to a total rotation of around $360^{\circ }$) in each category, as indicated in Figure 10. The total size of the dataset is 2088 oranges. In order to have constant size inputs to the network, all the maps created were scaled to 300 × 125 px (W × H). The difficulties in obtaining fruit with specific defects made the dataset imbalanced. The dataset was evenly split into training (75%), validation (15%), and test (10%) sets.

A human expert manually annotated the category of each example using the views as input. In the case that one orange had defects of more than one kind, the *predominant* damage was used to label the example. Notice that there may be an inevitable subjectivity in deciding which defect is the most important in some cases.

The maps were cropped in latitude to a value of $\lambda_{\max}=60$ degrees, and in longitude to a span of $360^{\circ }$. This way, all the maps had the same size. Data augmentation was carried out by vertical and horizontal mirroring.

Several CNN architectures were evaluated: DenseNet121 [23], ResNet50 [24], Inception-ResNet-V2 [25], and Xception [26].

Given that the dataset was imbalanced, with all classes equally important, the macro F1 score [27] (i.e., the arithmetic mean of all the per-class F1 scores) was used as a metric to assess the performance of the models.

As shown in Table 1, which summarizes the results obtained for the different architectures under consideration, the best result is obtained for the Resnet50 architecture, with a value of 0.68.

To achieve an idea of how good or bad this score is, we asked a second expert to classify the testing dataset into the same categories using the maps as the image to base the classification on. We compared this second manual classification with the ground truth generated by the first expert. Computing the resulting macro F1 score for the second expert yielded a value of 0.71.

Several results can be obtained from this experiment:

- The F1 score obtained with the CNN using the maps as input is close to the score of a human expert.
- Building a more balanced dataset should help improve the classifier’s performance.
- A certain amount of confusion between categories is expected, mainly due to several reasons, such as the possible co-existence of several defects in a significant number of cases. Since only one defect category is assigned to each example, one of them may be randomly selected as dominant.

## 4. Conclusions

This paper has presented a method to create maps of the surface of produce based on a collection of rotating views.

Analyzing one global image, the map offers various advantages over analyzing individual views. The most important one is that there is no need to combine the analysis results of the individual views to obtain a global classification of the fruit.

One of the essential contributions of this work is that the input for the method consists only of collections of views. No side information about rotation is needed. This fact facilitates the implementation of the method as a software extension on existing industrial inspection machines.

One important idea of the method is that every point in the map comes from two views at most, namely those where the point appears more centered on the view. This idea is crucial to achieving real-time performance. The whole process described in this paper, including geometry modeling and rotation estimation, can be carried out in less than 20 ms/fruit (for fruit with 16 views and using standard off-the-shelf processors).

Future work should address maps for fruit with geometric models other than those analyzed so far, particularly generic revolution geometric models such as avocados or pears.

## Figures and Tables

**Table 1:** F1 scores for the CNN architectures considered.

CNN Architecture	F1 Score
DenseNet	0.58
ResNet	0.68
Inception	0.54
Xception	0.56

## Data Availability

The data presented in this study are openly available in github at https://github.com/csdemeras/FSMap_complement/raw/main/supplement.pdf (accessed on 1 July 2022).
